# Purification and Thioflavin T Staining of *Bacillus* spp. Endospores for Fluorescence‐Based Assays

**DOI:** 10.1002/cpz1.70480

**Published:** 2026-10-05

**Authors:** Ephrem Debebe Zegeye, Marina Aspholm

**Affiliations:** ^1^ Department of Paraclinical Sciences, Faculty of Veterinary Medicine Norwegian University of Life Sciences (NMBU) Ås Norway

**Keywords:** *Bacillus* spp, endospore purification, endospores, fluorescent staining, thioflavin T

## Abstract

We present a simple, low‐cost, and efficient protocol for fluorescent staining of *Bacillus* spp. endospores using the thioflavin T (ThT) dye. The article provides step‐by‐step procedures for culturing *Bacillus paranthracis*, *Bacillus thuringiensis* (2 strains), and *Bacillus subtilis*, purifying endospores from pure cultures, and staining them with ThT. ThT‐stained endospores retain fluorescence for at least 2 years when stored at 4°C in the dark and remain fluorescent following formaldehyde fixation. Importantly, ThT staining does not impair spore viability, and stained endospores germinate normally. In contrast, vegetative *Bacillus* cells exhibit minimal to no ThT fluorescence. Overall, this robust and straightforward staining protocol is well suited for applications requiring fluorescently labeled endospores, including fluorescence microscopy, and provides a convenient plate‐based readout for germinant screening and related assays. © 2026 The Author(s). *Current Protocols* published by Wiley Periodicals LLC.

**Basic Protocol 1**: Endospore purification

**Basic Protocol 2**: Fluorescent staining of endospores

**Support Protocol**: Fluorescence microscopy of ThT‐stained spores

## INTRODUCTION

Endospores (spores) are highly resistant survival structures produced by certain bacteria within the phylum Firmicutes, most notably members of the orders *Bacillales* (e.g., *Bacillus*) and *Clostridiales* (e.g., *Clostridium*), in response to environmental stresses, e.g., nutrient limitation, desiccation, and other physiological or ecological pressures (Errington, 2003; Paredes‐Sabja et al., 2011). Through sporulation, these bacteria enter a dormant state that allows persistence under adverse environmental conditions for extended periods, and when the environment becomes conducive for growth, the spores germinate to give rise to vegetative cells (Moir & Cooper, 2015; Paredes‐Sabja et al., 2011). Spores are metabolically inactive and exhibit extraordinary resistance to environmental insults, including extreme heat, desiccation, toxic chemicals, freezing, and radiation at levels that would normally be lethal to vegetative cells (Nicholson et al., 2000). This resilience is attributed to their unique architecture, which incorporates multiple protective layers, a dehydrated core, and the accumulation of dipicolinic acid and small acid‐soluble proteins that stabilize nucleic acids (Nicholson et al., 2000; Setlow, 2014). A cross‐section of a bacterial spore reveals multiple distinct layers. From the inside out, these include the core, inner membrane, germ cell wall, cortex, outer membrane, and spore coat; in some species, e.g., *Bacillus cereus, Bacillus anthracis* and *Bacillus thuringiensis*, an additional thin, loose‐fitting layer called the exosporium is present (McKenney et al., 2013; Stewart, 2015).

Spore purification is a prerequisite for accurate and reproducible spore‐based assays as contaminating vegetative cells, cellular debris, and extracellular material can interfere with measurements and increase experimental variability. Effective purification removes these confounding components, thereby improving assay reliability and data interpretation. Importantly, spore purification is influenced by intrinsic properties of the spores themselves, including their surface features. Notably, spores of many *B. cereus* group species possess hair‐like surface structures known as endospore appendages (ENAs) (Pradhan et al., 2021; Sleutel et al., 2024; Zegeye et al., 2021). The ENAs significantly alter key physicochemical properties of the spores, including aggregation, sedimentation, and adhesion, which in turn affect purification behavior and efficiency (Albertsdottir Jonsmoen et al., 2025; Jonsmoen et al., 2024). As a result, ENAs directly influence the quality and reproducibility of purified spore preparations.

Fluorescent staining of endospores is an important tool for microscopic visualization in both research and diagnostic applications involving spore‐forming bacteria; however, the same structural features that confer resistance to environmental stressors also impede dye uptake compared with vegetative cells. In particular, the low permeability of the spore coat necessitates specialized staining approaches. For example, malachite green requires heat treatment to facilitate penetration into the spore. Moreover, the presence of an exosporium layer in certain *Bacillus* spp. (Stewart, 2015) represents an additional barrier that may further hinder effective staining.

Here, we describe a protocol for the purification of spores from *Bacillus* spp., followed by staining with the fluorescent dye thioflavin T (ThT). ThT is a benzothiazole dye widely used for detecting amyloid fibrils, owing to its strong fluorescence enhancement upon binding to β‐sheet–rich protein structures (Khurana et al., 2005; Vassar & Culling, 1959). In the context of bacterial spores, ThT binds efficiently to amyloid‐like proteins on the spore surface, resulting in a marked increase in fluorescence intensity compared to the weak emission of the free dye in solution (Xia et al., 2011). In Basic Protocol 1, four *Bacillus* species/strains are cultured on agar plates to sporulate, after which spores are harvested, washed in water, and finally purified using a Nycodenz density gradient (Coleman et al., 2007; Lindahl, 1995; Pradhan et al., 2021). In Basic Protocol 2, the purified spores are stained with the fluorescent dye ThT, following adaptations of previously published procedures (Xia et al., 2011).

*CAUTION*: Laboratory personnel must always wear lab coats, gloves, and safety glasses when handling *Bacillus* spp. Contaminated surfaces should be disinfected with sporicidal agents (e.g., sodium hypochlorite). Contaminated glassware must be sterilized by autoclaving prior to washing, and both solid and liquid waste should be autoclaved before disposal.

## Basic Protocol 1 SPORE PURIFICATION

In this protocol, *Bacillus* species of interest are cultured under sporulation‐promoting conditions until a high spore yield is achieved, after which the spores are harvested, and purified for ThT staining as described in Basic Protocol 2. *Bacillus* spp. can be grown either on agar plates (e.g., blood agar, LB agar, or other suitable agar medium) or in liquid (broth) media. Here, *B. paranthracis* and *B. thuringiensis* are cultivated on blood agar, whereas *B. subtilis* is grown on LB agar for 3 to 5 weeks. Extended incubation promotes spore maturation and minimizes residual vegetative cells. After the cell–spore mixture has been repeatedly washed with sterile water through cycles of centrifugation and resuspension, spores are then purified using a Nycodenz (5‐(N‐2,3‐dihydroxypropylacetamido)‐2,4,6‐tri‐iodo‐N,N'‐bis (2,3‐dihydroxypropyl)isophthalamide) density gradient. In this gradient, the relatively small and dense spores normally migrate to the bottom of the tube, whereas less dense cellular debris and vegetative cells remain in the upper layers, enabling efficient separation. As noted above, many species within the *B. cereus* group are decorated with ENAs, which may necessitate optimization of centrifugation speeds during both washing and density gradient centrifugation.

### Materials

*Bacillus* spp.:
*B. paranthracis* NVH 0075/95 (Lund & Granum, 1996; Pradhan et al., 2021)
*B. paranthracis* ∆*ena1*ABC/∆*ena*3A (S‐ and L‐ENA‐depleted strain) (Sleutel et al., 2024)
*B. paranthracis* ∆*ena1B*::p*enaAB* (Pradhan et al., 2021), a strain that produces longer and more abundant S‐ENA (hereafter referred to as S‐ENA‐overexpressing strain)
*B. thuringiensis* 407 (Sleutel et al., 2025)
*B. thuringiensis* 407 ∆*f‐ena* (F‐ENA depleted strain) (Sleutel et al., 2025)
*B. thuringiensis* (subsp. *Kurstaki*) strain (GEVI1716, acquired from Prof. Henri De Greve's lab, VUB, Belgium).
*B. thuringiensis* (subsp. *Kurstaki*) ∆*f‐ena* (F‐ENA depleted strain; from Prof. Marina Aspholm's lab)
*B. subtilis* 507 (from Prof. Marina Aspholm's lab)
Blood agar plates (see recipe)
LB agar plates (see recipe)
dH_2_O, 4°C, sterile, 2 L
Immersion oil (e.g., Leica TypN immersion oil; cat. no. 11513860)
Nycodenz solutions (20%, 40% and 47%) (see recipe)

BSL‐2 safety cabinet (e.g., ninoSAFE Class II 1200)
1‐µl inoculating loop (VWR, cat. no. 612‐5694)
30° and 37°C incubators (e.g., FRIOCELL 222‐Eco line)
Standard microscope slides (e.g., Epredia microscope slides, Fisher Scientific, cat. no. 12342108)
Coverslip, 22 × 22–mm (VWR, cat. no. 631‐1570)
Phase‐contrast microscope (e.g., Olympus BX51 equipped with a 100× phase contrast objective)
10‐µl inoculating loop (Fisher Scientific, cat. no. 12870155)
15‐ml conical centrifuge tubes (Thermo Fisher Scientific, cat. no. 339650); preferably low binding (USA scientific, cat. no. 4011‐2216)
Vortex (e.g., Stuart, cat. no. SA8)
Centrifuge (e.g., Thermo Fisher Scientific, Heraeus Megafuge 16R), 4°C
Sterile swabs (Sarstedt, cat. no. 80.626)
100‐ to 1000‐µl pipette and tips (preferably low binding pipette tips, e.g., Sigma‐Aldrich, cat. no. CLS4140‐768EA)
5‐ml pipette and tips, e.g., Finnpipette (Labsystems, cat. no. J90141, 4500) and Finntip 5 ml (Thermo Fisher Scientific, cat. no. 9402030)

*CAUTION*: Perform all inoculations in a BSL‐2 biosafety cabinet using strict aseptic techniques.

#### Culturing *Bacillus* spp. for sporulation

1 Streak the *Bacillus* spp. of interest from a −80°C glycerol stock onto a blood agar or LB agar plate using a 1‐µl inoculating loop. Incubate the plate upside down overnight at 30° or 37°C, depending on optimal growth temperature of the strain.
2 The following day, pick a single colony and streak it onto a fresh blood agar or LB agar plate, spreading the inoculum over the entire plate surface.
3 Place the plates inside a plastic bag and incubate them inverted for 3 to 5 weeks at the species‐specific optimal temperature for sporulation. The plastic bag prevents the plates from drying out and reduces the risk of contamination.
  As an alternative to cultivation on solid media (and for more rapid spore production in B. cereus group spp.), inoculate a single colony into 10 ml of BHI medium and incubate overnight at 30° or 37°C with shaking at 200 rpm. The following day, transfer 1 ml of the overnight culture per 0.5 L of sporulation medium [8 g/L Bacto nutrient broth (Difco), 1 µM FeSO_4_, 2.5 µM CuCl_2_, 12.5 µM ZnCl_2_, 66 µM MnSO_4_, 1 mM MgCl_2_, 5 mM (NH_4_)_2_SO_4_, 2.5 µM Na_2_MoO_4_, 2.5 µM CoCl_2_, 1 mM Ca(NO_3_)_2_, pH 7.6] (Pradhan et al., 2021) in a 2‐L Erlenmeyer flask. Incubate for up to 1 week with shaking, while periodically monitoring the progress of sporulation by analyzing samples using phase‐contrast microscopy (see step 5 and Fig. 1A). Sporulation is typically completed within 2 days for B. paranthracis NVH 0075/95. Once sporulation is complete, harvest cells by centrifugation as described in step 8. A limitation of the liquid sporulation medium is that it yields a higher proportion of phase‐dark spores (e.g., see Fig. 1B) compared with sporulation on blood agar plates for B. paranthracis NVH 0075/95.

**Figure 1:**
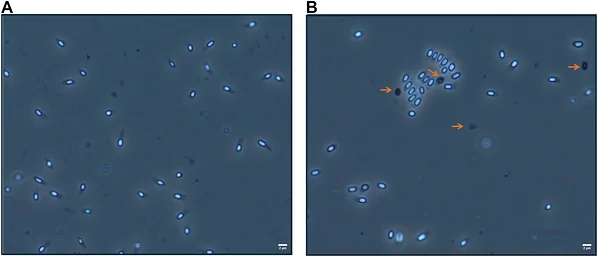
Phase‐contrast micrographs of (**A**) *B. thuringiensis* 407 spores and (**B**) *B. thuringiensis* (subsp. *kurstaki*). Orange arrows in (**B**) indicate phase‐dark spores.

#### Monitoring sporulation

4 After ∼3 weeks, monitor sporulation by gently scraping a small sample from the agar surface with a sterile 1‐µl inoculating loop and mixing it with a drop of sterile water on a microscope slide. Apply a coverslip.
5 Examine the preparation by phase‐contrast microscope with a 100× oil‐immersion objective (see Fig. 1 for examples of phase‐bright spores). If a high proportion of intact vegetative cells and/or spores still retained within the mother cells is observed, continue incubation for an additional 1 to 2 weeks before harvesting.

#### Harvesting spores

6 Once sporulation is complete:
  a. Gently scrape the spores and residual cells from the agar surface using a 10‐µl inoculating loop or a bent glass rod.
  b. Transfer the collected biomass into a 15‐ml conical tube containing 5 to 7 ml of sterile distilled water.
    A single 15‐ml tube is typically sufficient for cells harvested from two agar plates. If additional material is required, multiple 15‐ml tubes may be processed in parallel.
  c. After harvesting, adjust the final volume to 10 ml with sterile distilled water.
7 Vortex the suspension thoroughly to disrupt clumps and disperse the spores and cell debris evenly.
8 Centrifuge the suspension for 10 min at 2000 to 4500 × *g* (depending on the strain), 4°C. If a visible pellet does not form or the pellet remains loose, increase the centrifugation speed stepwise (e.g., 2000, 2500, 3000 × *g*, etc.) until a compact pellet is obtained.

#### Washing spores and enrich for spore dominating layer

9 Carefully discard the supernatant. Cell debris often adheres to the tube wall (see Supporting Information, Fig. S1); remove it with a sterile swab.
10 Resuspend the pellet in a final volume of 10 ml of sterile distilled water and centrifuge as described in step 8. Repeat this wash step two additional times.
11 After each centrifugation, if two or more distinct layers are visible (see Fig. 2A), sample a small portion using 1‐µl inoculating loop, and examine it by phase‐contrast microscopy. Carefully remove the layer(s) enriched in cell debris by pipetting, leaving behind the relatively clean spore pellet.
  During centrifugation, distinct pellet layers are often observed (Fig. 2A): a dark, debris‐rich upper layer (Fig. 2A‐B) and a lighter brown lower layer, particularly when cultures are grown on blood agar (Fig. 2). It is recommended to examine each layer by phase‐contrast microscopy to determine which layer predominantly contains spores, as this can vary between Bacillus spp. Additionally, ENAs present in some strains may influence sedimentation rates and affect pellet separation, as mentioned above.

**Figure 2:**
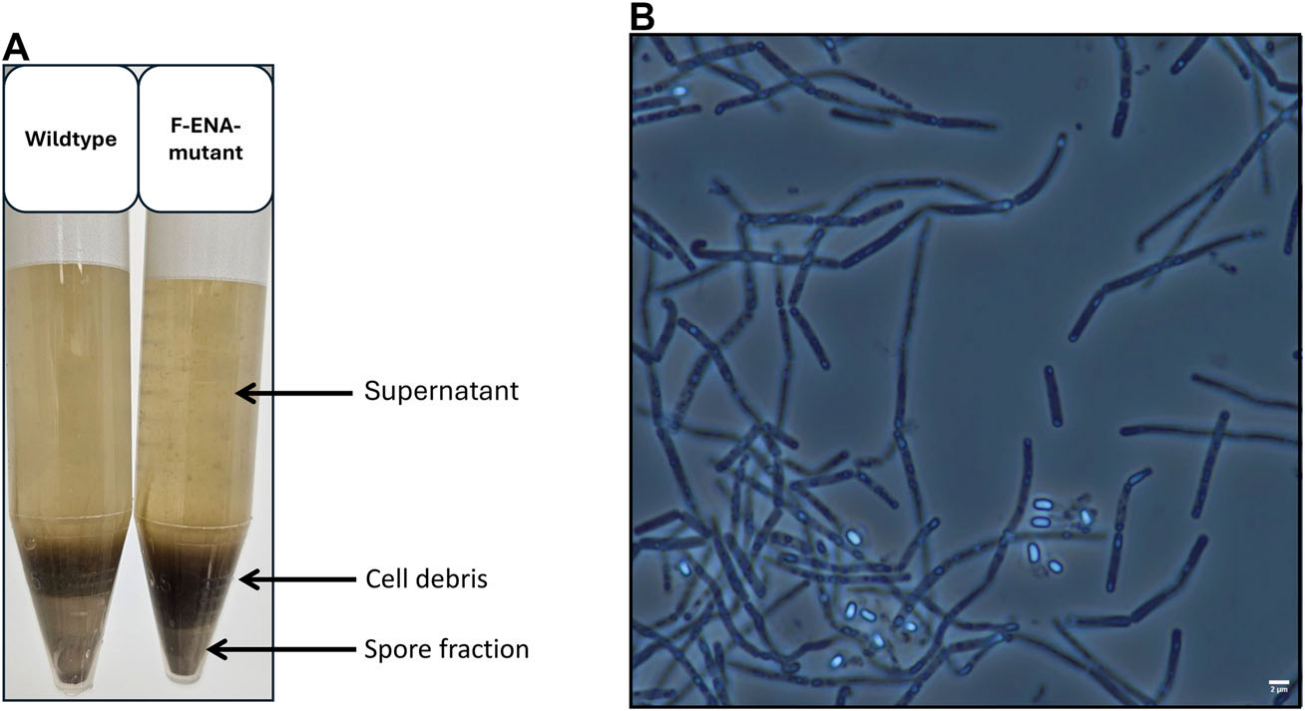
(**A**) Distinct layers formed during washing steps of spores harvested from blood agar plates. The image shows purification of spores of *B. thuringiensis* (subsp. *kurstaki*) wildtype and F‐ENA mutant. (**B**) Phase‐contrast micrograph of the upper dark brown layer of *B. paranthracis* spore purification, showing vegetative cells and cell debris.

12 After the final enrichment wash, resuspend the pellet in 3 ml of 20% Nycodenz solution. When pipetting, carefully avoid touching the tube walls to minimize contamination from residual debris (see Supporting Information, Fig. S1) adhering to the sides.

#### Density gradient purification

13 In a sterile 15‐ml conical tube, prepare a Nycodenz gradient by carefully layering 4 ml of 47% Nycodenz (bottom layer) and 4 ml of 40% Nycodenz (middle layer) (see Fig. 3A). Tilt the tube slightly before dispensing the 40% Nycodenz gently along the tube wall to minimize mixing.

**Figure 3:**
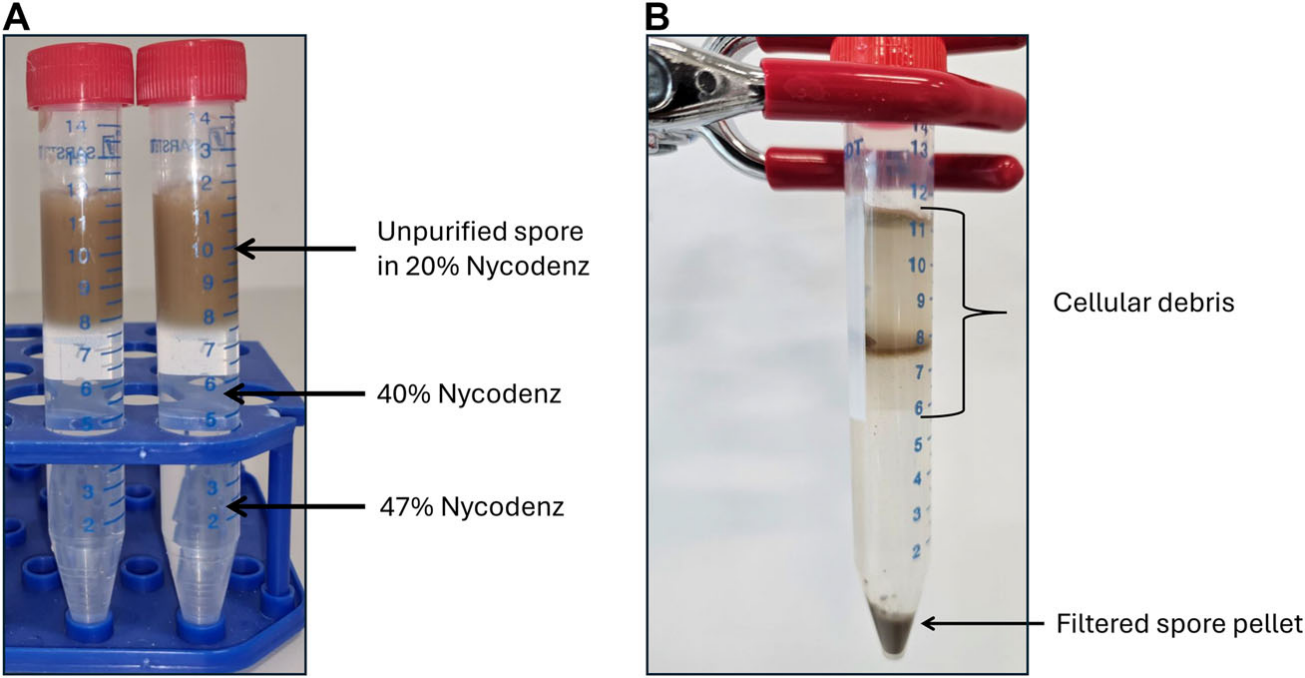
Nycodenz density gradient *B. paranthracis* spore purification before (**A**) and after (**B**) centrifugation.

14 Gently overlay the spore suspension in 20% Nycodenz (step 12) onto the top of the Nycodenz gradient. Take care not to disturb the underlying layers (see Fig. 3A). Tilt the tube and apply the suspension gently along the tube wall to minimize mixing between the layers.
15 Centrifuge the gradient 45 min at 4500 × *g*, 4°C.
  For the B. thuringiensis strains and B. subtilis, a higher speed (up to 5300 × g) was required.
16 Carefully remove and discard the Nycodenz supernatant by pipetting without disturbing the pellet at the bottom of the tube (Fig. 3B).
17 Resuspend the purified spore pellet in 1 ml of sterile water and transfer to a fresh tube. During transfer, avoid touching the tube walls, as they may contain cell debris.
18 Add sterile distilled water to a final volume of 10 ml, vortex, and centrifuge 10 min at 2000 to 4500 × *g* (depending on the strain), 4°C.
19 Wash the pellet two more times with sterile water to remove any residual Nycodenz.
20 Resuspend the purified spore in 1 to 3 ml of sterile distilled water.
21 Verify spore purity by examining a sample by a phase‐contrast microscopy using a 100× phase‐contrast objective.
22 Store the purified spores at 4°C until staining is carried out as described in Basic Protocol 2.
  Note that purified Bacillus spp. spores are stable for at least 3 to 6 months at 4°C in sterile distilled water.

The step‐by‐step procedure described in Basic Protocol 1 is illustrated in Figure 4.

**Figure 4:**
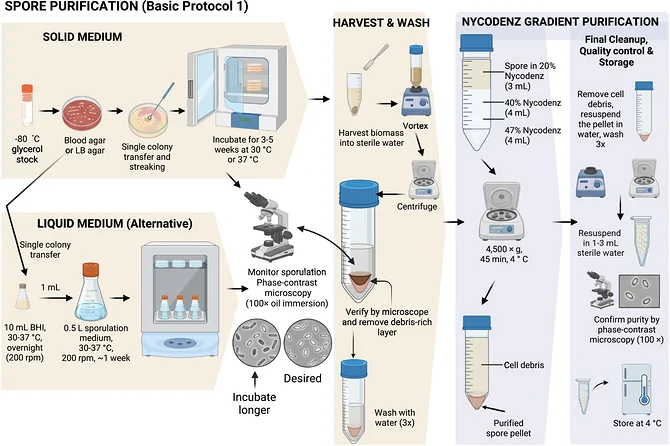
Schematic workflow for cultivation, sporulation, and purification of *Bacillus* spp. spores (Basic Protocol 1). Illustrations were created using BioRender.

## Basic Protocol 2 FLUORESCENT STAINING OF *BACILLUS* SPORES

In this protocol, purified spores (from Basic Protocol 1) are stained with the fluorescent dye ThT, as illustrated in Figure 5. Briefly, purified spores (step 20) are centrifuged to remove the resuspension medium. Based on the measured weight of the spore pellet, 8 ml of 1 mM ThT is added per g of purified spores, corresponding to 8 µmol ThT per g of spore weight. The mixture is incubated in the dark with gentle shaking for 24 hr at room temperature. Following incubation, samples are centrifuged to remove excess dye and rinsed with water. Stained spores are examined using phase‐contrast fluorescence microscopy. Since ThT has an excitation maximum at 440 to 450 nm and an emission maximum at 480 to 490 nm, imaging is best performed with a cyan fluorescent protein (CFP) filter, with fluorescein isothiocyanate (FITC) serving as an alternative.

**Figure 5:**
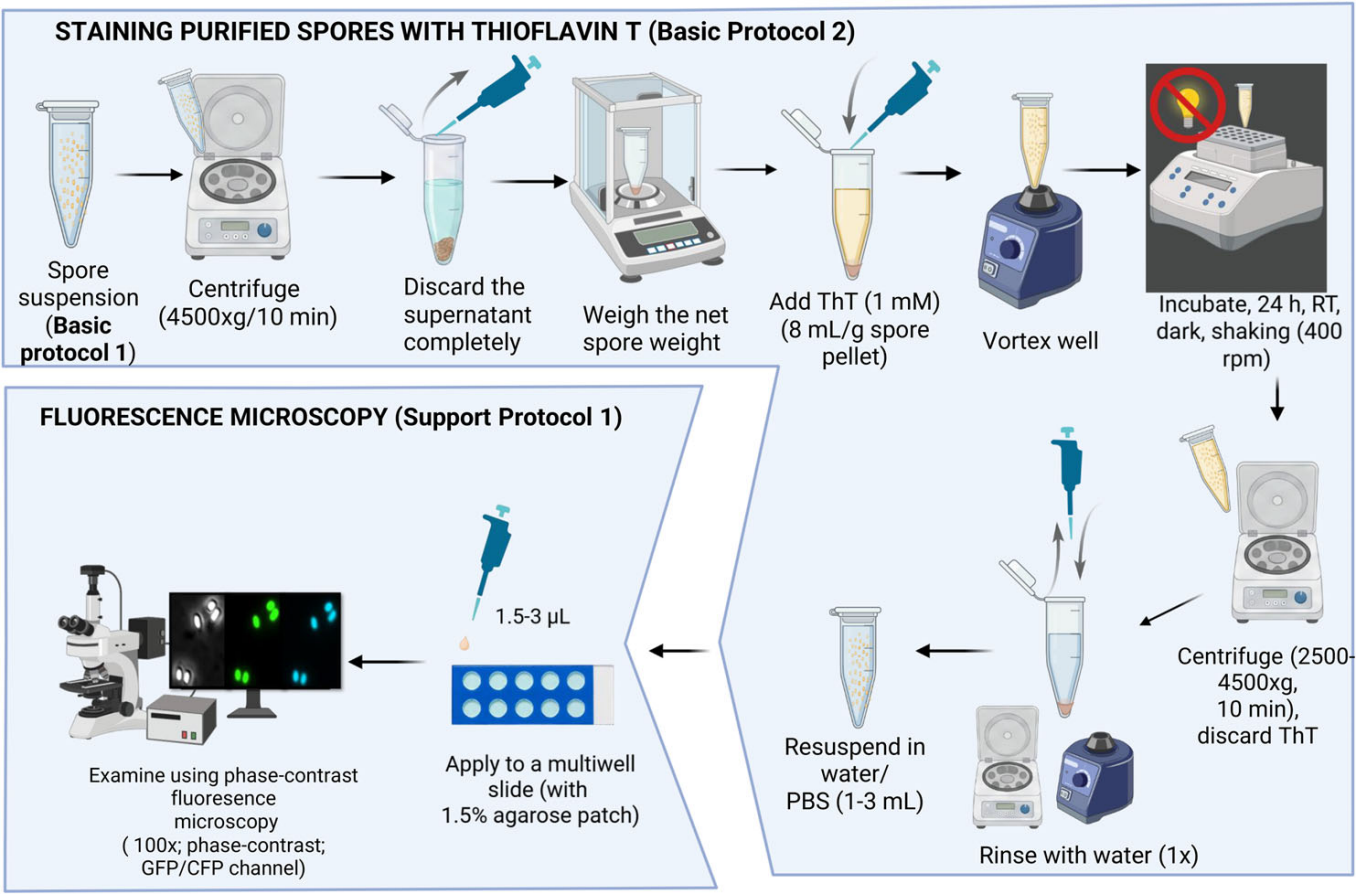
Schematic workflow for ThT staining of *Bacillus* spp. spores and fluorescence microscopy imaging (Basic Protocol 2). Illustrations were created using BioRender.

*CAUTION*: When working with ThT and methanol, always wear appropriate protective equipment, including gloves, a lab coat, and safety glasses. Avoid inhaling ThT powder. Perform all powder handling and dissolving ThT in methanol inside a fume hood. If available, use a dedicated powder‐handling or balance enclosure for added safety. Methanol is flammable and needs to be handled with caution. Dispose of chemical waste according to your institution's regulations and disinfect contaminated surfaces with a sporicidal agent such as sodium hypochlorite.

### Materials

1 mM ThT working solution (see recipe)
dH_2_O, sterile

2‐ml microtube, preferably graduated (e.g., Sarstedt, cat. no. 72.694.106)
Microtube rack (e.g., Fisher Scientific, cat. no. 11968074)
Analytical balance (e.g., Mettler Toledo, Balance XSR304)
100‐ to 1000‐µl pipette and tips, preferably low binding (e.g., Sigma‐Aldrich, cat. no. CLS4140‐768EA)
Centrifuge (e.g., Thermo Fisher Scientific, Heraeus Megafuge 16R)
Orbital shaker (e.g., Eppendorf, ThermoMixer C)

#### ThT staining

1 Take a new sterile tube and record the weight of the empty tube using an analytical balance. Pipette the spore suspension to be stained (Basic Protocol 1, step 20) into the tube.
2 Centrifuge the purified spore suspension 10 min at 4500 × *g*, room temperature.
  For strains that do not form a compact pellet (e.g., strains overexpressing S‐ENAs), the centrifugation speed may be increased to 5300 × g.
3 Carefully remove the supernatant and centrifuge again 5 min at 4500 × *g*, room temperature.
4 Remove the supernatant completely and measure the weight of the tube containing the spore pellet.
5 Calculate the net spore pellet weight by subtracting the empty tube weight (step 1) from the total weight.
6 Add 8 ml of 1 mM ThT per g of spore pellet (equivalent to 8 µmol ThT per g of spore). Mix thoroughly by vortexing or pipetting up and down.
7 Incubate the mixture in the dark at room temperature for 24 hr with gentle mixing (e.g., at 400 rpm using ThermoMixer C, Eppendorf).
  Alternatively, rapid staining can be performed by heating the spore‐ThT mixture at 80°C for 10 min; however, a substantial proportion of spores may become phase‐dark (Fig. 1B) upon storage.
8 Centrifuge the sample 10 min at 2000 to 4500 × *g* (depending on the ability of the strain to form a compact pellet), room temperature, to remove excess ThT.
9 Carefully discard the supernatant and rinse the pellet with sterile distilled water using a volume twice that of the ThT solution used.
10 Centrifuge 10 min at 2000 to 4500 × *g*, room temperature, and discard the supernatant.
11 Resuspend the pellet in an appropriate volume of sterile water or phosphate‐buffered saline (PBS; Current Protocols, 2006), depending on downstream applications.
  Note that Bacillus spores are often hydrophobic and may adhere to tube walls or pipette tips (Albertsdottir Jonsmoen et al., 2025). When possible, the use of low‐binding tubes and pipette tips is recommended, particularly when accurate spore concentrations are critical for downstream applications and after colony forming unit (CFU) estimations have been performed.

## FLUORESCENCE MICROSCOPY OF ThT‐STAINED SPORES

In this protocol, ThT‐stained spores and unstained control spores are examined using a fluorescence microscopy to assess the success of staining. Spores are immobilized on an agarose patch prepared on a microscope slide, after which a coverslip is applied. Samples are imaged using a fluorescence microscope equipped with a phase‐contrast objective, which allows clear visualization of spores based on differences in refractive index. ThT has an excitation maximum of 440 to 450 nm and an emission maximum of 480 to 490 nm; therefore, it is better visualized using a CFP filter set than an FITC filter set.

### Materials

1.5% agarose (see recipe)
ThT‐stained spores (see Basic Protocol 2)
Immersion oil (e.g., Leica TypN immersion oil; cat. no. 11513860)

Microwave oven or heat block
100‐ to 1000‐µl pipette and tips, preferably low binding (e.g., Sigma‐Aldrich, cat. no. CLS4140‐768EA)
Multitest slide, 10‐well (MP Biomedicals, cat. no. 096041805E)
Standard microscope slides (e.g., Epredia microscope slides, Fisher Scientific, cat. no. 12342108)
Scalpel
BSL‐2 safety cabinet (e.g., ninoSAFE Class II 1200)
Coverslip (24 × 60–mm) (VWR, cat. no. 631‐1575)
Fluorescence microscope equipped with a 100× phase‐contrast objective and a CFP or FITC/GFP filter set (e.g., Nikon Eclipse Ti2)

#### Preparation for microscopy of ThT‐stained *Bacillus* spp. spores

1 Melt the 1.5% agarose by briefly warming it in a microwave oven or on a heating block until it is completely liquefied.
2 Pipette 1 ml of molten agarose and dispense ∼100 µl into each well of a Multitest slide. Immediately place a clean microscope slide on top, avoiding air bubbles, and press gently to form a flat agarose layer.
3 Allow the agarose to solidify on the bench for ∼30 min. If not used immediately, store up to 24 hr at 4°C to prevent over‐drying.
4 To apply the spore sample, remove the covering slide by gently sliding it sideways. Trim away any excess agarose at the edges with a scalpel to prevent tearing of the thin agarose pad.
5 Apply 1.5 to 3 µl of appropriately diluted, ThT‐stained spores to the agarose surface and tilt the slide slightly to let the suspension spread evenly. Include unstained spore sample(s) as control(s). Each Multitest slide can be used for up to 10 samples.
6 Allow the sample to adsorb onto the agarose surface by incubating the slide in a BSL‐2 cabinet for 1 to 2 min with the airflow on.
7 Place a coverslip over the Multitest slide and press very gently to ensure even contact with the agarose layer.

#### Fluorescence microscopy

8 Apply immersion oil to the 100× objective (or to the coverslip, depending on the microscope configuration). Mount the slide upside down on the stage of the inverted microscope.
9 Using the coarse focus adjustment, raise the objective until it makes contact with the immersion oil.
10 Switch to the fine adjustment to bring the spore samples into sharp focus.
11 Scan the field of view and identify regions where individual spores are clearly resolved and well separated.
12 Select the appropriate imaging modes, including phase‐contrast and fluorescence channels (CFP or FITC/GFP, as required).
13 Adjust imaging and exposure settings for each channel to achieve clear visualization of spores while maintaining appropriate signal levels.
  To begin with, the following exposure settings may be used: phase contrast, 1 s; CFP, 30 ms or FITC, 1 s.
14 Capture images and evaluate the signal intensity. If the signal is too weak, increase the exposure time; if signal is too strong or saturated, decrease the exposure time.
15 Save the captured images.

## REAGENTS AND SOLUTIONS

### Agarose, 1.5% (w/v)

For 50 ml, weigh 0.75 g agarose (Sigma‐Aldrich, cat. no. A9539; 9012‐36‐6) and add distilled water to a final volume of 50 ml. Heat the mixture in a microwave, mixing occasionally, until the agarose is completely dissolved. Complete dissolution is essential to prevent undissolved particles, which can increase background signals and reduce image quality during fluorescence imaging. Store up to 3 months at room temperature.

### Blood agar

Dissolve 40 g of Blood agar base No. 2 (Oxoid, cat. no. CM0271) in distilled water to a final volume of 1 L, heat the mixture until it is completely dissolved, then sterilize it by autoclaving at 121°C for 15 min. After cooling the medium to 45° to 50°C, mix in sterile blood to reach a final concentration of 5% (v/v). Dispense ∼20 ml into each 9‐cm Petri dish (HEGER AS, cat. no. 1065) and allow the agar to solidify. Store plates up to 1 month at 4°C.

### LB agar

Prepare LB agar by dissolving 37 g of LB agar powder (Merck, cat. no. 110283) in 1 L of distilled water. Heat with stirring until fully liquefied, then sterilize by autoclaving at 121°C for 15 min. Dispense ∼20 ml into each 9‐cm Petri dish (HEGER AS, cat. no. 1065) and allow to solidify. Store plates up to 2 months at 4°C.

### Nycodenz solutions, 20%, 40%, and 47% (w/v)

For 50 ml, prepare 20%, 40%, and 47% Nycodenz solutions by dissolving 10 g, 20 g, and 23.5 g of Nycodenz (Serumwerk Bernburg AG, cat. no. 18003), respectively, in 40 ml of distilled water. Once fully dissolved adjust each solution to a final volume of 50 ml. Sterilize by autoclaving at 121°C or by filtration through a 0.22‐µm filter. Store in the dark up to 1 year at 4°C.

### Thioflavin T (ThT) stock solution

For 1 ml, weigh 100 mg of thioflavin T (Merck, cat. no. T3516) and transfer it to a graduated microcentrifuge tube. Add ∼750 µl of pure methanol (Merck, cat. no. 67‐56‐1) and vortex until fully dissolved. Adjust the final volume to 1000 µl using the graduation on the tube to obtain a stock solution with a concentration of ∼314 mM. Seal the tube with Parafilm to avoid evaporation, wrap in aluminum foil to protect from light, and store up to 1 month at 4°C.

### Thioflavin T, 1 mM

For 20 ml, immediately before staining, prepare a 1 mM working solution by mixing 63.7 µl of ThT stock solution (∼314 mM; see recipe) with 2 mM Tris, pH 8.5 (see recipe), and adjusting the final volume to 20 ml. Mix thoroughly by vortexing. Protect the solution from light. Prepare immediately before use.

### Tris·HCl, pH 8.5, 1 M

For 50 ml, weigh 6.06 g of Tris (hydroxymethyl aminomethane) (Merck, cat. no. 77‐86‐1) and dissolve it in 30 ml of deionized water. While stirring, adjust the pH to 8.5 using 2 M HCl. Finally, bring the volume to 50 ml with deionized water. Sterilize by autoclaving at 121°C or by filtration through a 0.22‐µm filter. Store up to 1 year at room temperature.

### Tris·HCl, pH 8.5, 2 mM

For 50 ml, pipette 100 µl of 1 M Tris·HCl stock solution (see recipe) into a volumetric container and bring the final volume to 50 ml with distilled water. Mix thoroughly. Sterilize by autoclaving at 121°C or by filtration through a 0.22‐µm filter. Store up to 1 month at room temperature.

## COMMENTARY

### Background Information

ThT and other fluorescent dyes have been used to stain and characterize endospores including safranin O, auramine O, rhodamine B, Congo red (Kuwana et al., 2025) and acridine orange (Bruno, 2015). In *B. subtilis*, ThT, Congo red and safranin O (red) primarily stain the inner spore coat, whereas rhodamine B and safranin O (green) stain the cortex and the basement layer of spore coat (Kuwana et al., 2025). ThT is a benzothiazole dye widely used for staining amyloid fibrils due to its strong fluorescence enhancement upon binding to β‐sheet–rich protein structures (Khurana et al., 2005; Vassar & Culling, 1959). In aqueous environments, ThT has been shown to form micelles at concentrations commonly used in fluorescence assays for monitoring fibril formation (10 to 20 µM) (Khurana et al., 2005). Xia et al. (2011) were the first to report the application of ThT staining to endospores. They demonstrated that ThT binds efficiently to spores, resulting in a marked increase in fluorescence compared to the weak signal of free dye in solution. This enhanced fluorescence is attributed both to the strong accumulation of dye within the spore surface layers and to the increased quantum yield of ThT upon binding (Xia et al., 2011). Notably, ThT staining is non‐destructive and does not interfere with spore germination, enabling its use in live‐cell assays. During germination, ThT initially accumulates outside the spore core, but subsequently penetrates into spore core as germination proceeds (Li et al., 2023)

The advantages of ThT staining of endospores include a simple staining procedure, rapid labeling (as short as 10 min), low cost, high contrast, and the absence of harsh pretreatment steps (Xia et al., 2011). Studies suggest that ThT preferentially stains mature spores and early log phase cells compared to its analogs APBT and BTA‐1 (Kuwana et al., 2023). Free ThT exhibits an excitation maximum at ∼385 nm, whereas bound dye shows an excitation maximum at ∼450 nm (LeVine, 1993). In line with this, the emission maximum is ∼445 nm for the free dye and ∼482 nm for the bound form (LeVine, 1993). ThT‐stained spores are best visualized using a CFP filter set, as its excitation and emission wavelengths closely match the spectral properties of ThT, ensuring strong and specific fluorescence (LeVine, 1993).

### Critical Parameters

#### Basic Protocol 1

It is important to grow each strain under appropriate sporulation conditions to obtain spores of sufficient quality and yield. These conditions, including the choice of growth medium, incubation temperature, and the duration required for complete release of spores from the mother cells, are strain dependent. During washing and spore enrichment steps, care should be taken to collect the fraction predominantly containing spores. The density‐gradient purification step is also critical for both spore quality and yield; therefore, centrifugation speed should be carefully optimized to maximize recovery and purity.

#### Basic Protocol 2

To achieve consistent staining and minimize batch‐to‐batch variation, it is important to accurately measure the weight of the purified spore pellet. ThT working solutions should be prepared fresh, and thorough mixing of spores with ThT during the staining period is essential to ensure uniform staining.

### Troubleshooting

Common problems encountered during spore purification and ThT staining, as well as their possible causes and potential solutions, are summarized in Table 1 to aid troubleshooting and optimization of the procedures.

**Table 1:** Troubleshooting Guide for *Bacillus* Spore Purification and ThT Staining

Problem	Possible cause	Potential solution
Predominantly unreleased spores in the mother cell	Insufficient time given for completion of sporulation	Extend the incubation period to allow sporulation to be completed
	Sporulation conditions are not optimal	Optimize sporulation conditions: culturing medium, pH, temperature, and oxygen
Low purity in spore preparation	Poor enrichment of spores during the washing and/or failure of density gradient step	Enrich the spore‐dominant layer by repeated washing and centrifugation, followed by removal of the cell debris layer; alternatively, try alternative gradient purification methods, e.g., as described by Coleman et al. (2007) or 50% sucrose gradient purification (Weldy et al., 2020)
Low spore yield	Poor sporulation conditions	Optimize sporulation conditions by adjusting the culture medium, incubation temperature, and incubation duration; begin with a sufficiently concentrated cell/spore sample
	Loss of spores during density‐gradient purification (spores stuck in the Nycodenz solution in the middle)	Increase the centrifugation speed if spores fail to pass through the Nycodenz density gradient; if this does not resolve the issue, omit the density‐gradient purification step and instead rely on repeated spore‐enrichment procedures (Basic Protocol 1, step 11‐12)
Presence of ThT unstained spores	Possible spore clumps and spore‐ThT not mixed well	Ensure that the spore‐ThT mixture is thoroughly mixed by vortexing or pipetting until no visible clumps remain; maintain continuous mixing throughout the staining procedure to prevent settling or clumping; if the issue persists, consider titrating the ThT volume and concentration

### Understanding Results

In this protocol, *B. paranthracis*, *B. thuringiensis* 407, and *B. thuringiensis* subsp. *kurstaki*, along with their corresponding ENA knockout mutants (see Supporting Information, Table S1), were cultured on blood agar plates. *B. subtilis*, which lacks an exosporium, was cultured on LB agar and included for comparison. Additionally, a *B. paranthracis* strain producing elongated S‐ENAs (∆*ena1B*::*penaAB)* (Pradhan et al., 2021) was included to assess the effect of ENAs on spore purification. Cultures were incubated for ∼4 weeks to allow complete sporulation. Completion of sporulation was verified by taking a small sample with a 1‐µl inoculating loop, suspending it in 10 µl of sterile distilled water on a microscope slide, placing a coverslip and examining the preparation using a 100× phase‐contrast objective (Fig. 1). All *Bacillus* spp. examined (*B. paranthracis*, *B. subtilis*, and *B. thuringiensis*) showed efficient sporulation after 4 weeks of incubation, and no further incubation was required.

The limitation of using solid media is the extended time required for spore formation, which can take several weeks before sufficient spores are available for purification. As an alternative, liquid media, such as sporulation medium (Pradhan et al., 2021) can be used to produce spore biomass within a few days. Indeed, the *B. paranthracis* NVH 0075/95 strain we tested completed sporulation in <36 hr in this medium (Zegeye et al., 2026). However, we observed that this approach may result in a higher proportion of phase‐dark spores (Fig. 1B) in *B. paranthracis*, compared with growth on blood agar plates. Even when using solid media, medium composition influences spore quality; for *B. subtilis*, cultures grown on blood agar plates produced more phase‐dark spores than those grown on LB agar. Consequently, in this article, we proceeded with *B. subtilis* spore samples derived from LB agar cultures.

Biomass harvested from two agar plates was transferred to a 15‐ml tube and resuspended by vortexing. The final volume was adjusted to 10 ml, and the samples were centrifuged for 10 min at 4°C. For *B. paranthracis* wild‐type and ENA mutant strains, centrifugation at 1500 to 2000 × *g* was sufficient to pellet the spores. In contrast, the S‐ENA–overexpressing strain (Pradhan et al., 2021) did not pellet effectively even at 2500 × *g* and required centrifugation at 3500 × *g* to achieve adequate sedimentation, indicating that ENAs influence spore pelleting efficiency. In addition to ENAs, other spore properties, such as size and density, are also likely to affect sedimentation behavior. For example, the *B. subtilis* str. 507 required an even higher centrifugation speed (4500 × *g*) to pellet spores effectively, despite the absence of ENAs in this species. These observations highlight the need to empirically determine optimal centrifugation conditions for each strain.

During the spore washing and enrichment steps, two distinct layers were observed following centrifugation (Fig. 2A). In most samples, this separation was evident after the first wash, whereas in others (e.g., *B. thuringiensis* 407) it became apparent only after the second wash (Fig. 2A). Typically, strains grown on blood agar produced a gray spore‐rich fraction that settled at the bottom of the tube, while a dark brown, vegetative cell–rich layer remained at the top (Fig. 2B). After confirming that the lower fraction contained spores, the upper layer, composed primarily of cell debris, was carefully removed by pipetting after each resuspension and wash step to enrich the spore fraction. In *B. subtilis*, no such distinct layering was observed, presumably because growth on LB agar yields a whitish biomass compared with the darker biomass obtained from blood agar plates.

During the Nycodenz density gradient step, centrifugation at 4500 × *g* was sufficient for all strains tested except the S‐ENA–overexpressing *B. paranthracis* strain and *B. subtilis*, which required centrifugation at 5300 × *g*. After centrifugation for 45 min, two distinct layers were typically observed, although three layers were present in some cases (Fig. 3B). The bottom layer corresponded to purified spores, whereas the upper layers consisted mainly of cellular debris and spores trapped within the cellular matrix. The relative thickness of these layers varied among species, and even between wild‐type and ENA mutant strains, likely reflecting differences in the presence or absence of ENAs (Pradhan et al., 2021; Sleutel et al., 2024, 2025), as shown in Supporting Information (Fig. S2). Indeed, in wild‐type *B. thuringiensis* 407, a thicker layer in the middle was observed compared with the corresponding mutant (see Supporting Information, Fig. S2), indicating that the presence of F‐ENAs (Sleutel et al., 2025) influences purification of spores. In *B. subtilis*, only two layers, one at the bottom and one in the middle, were observed. The yield obtained from two agar plates following the purification protocol is presented in Table 2.

**Table 2:** Spore Yield Following Nycodenz Gradient Purification and ThT Staining

*Bacillus* spp. (wild type and mutants)	Net spore weight obtained (mg)	Volume of 1 mM ThT added (µl)
*B. paranthracis* NVH 0075/95	81.4	651
*B. thuringiensis* 407	18.2	146
*B. thuringiensis* (subsp. *kurstaki*)	14.8	118
*B. subtilis* 507	12.4	99
*B. paranthracis* ∆*ena1ABC/*∆*ena3A*	94.4	755
*B. paranthracis* ∆*ena1B*::p*enaAB*	17.6	141
*B. thuringiensis* 407 ∆*f‐ena*	32.7	262
*B. thuringiensis* (subsp. *kurstaki*) ∆*f‐ena*	6.6	53

As shown in Figure 6 and Figure 7, *B. subtilis* and both wild‐type and ENA mutant strains of *B. paranthracis* and *B. thuringiensis* were successfully stained with 1 mM thioflavin T (ThT) following Basic Protocol 2. Little to no excess ThT fluorescence was observed in the supernatant, and, importantly, no unstained spores were observed in any of the samples (Fig. 6 and Fig. 7). Notably, vegetative cell debris showed weak or no ThT staining (Fig. 6A). Furthermore, toxin crystals in *B. thuringiensis* subsp. *kurstaki* were not labeled by ThT (Fig. 6C and Fig. 7D), indicating that ThT specifically stains spores. Mean fluorescence intensity values obtained for individual spores are presented in Figure 8. Notably, the median fluorescence intensity differed among the three strains examined. These differences may reflect intrinsic variation in spore surface composition as well as variation in preparation purity. Unstained spores exhibited minimal autofluorescence under identical imaging conditions (see Supporting Information, Fig. S6). In *B. thuringiensis* for example, the fluorescence intensity of the unstained spores was ∼97% lower than that of the stained spores, indicating that autofluorescence contributed only ∼3% of the signal detected in the stained spores (see Supporting Information, Fig. S6D).

**Figure 6:**
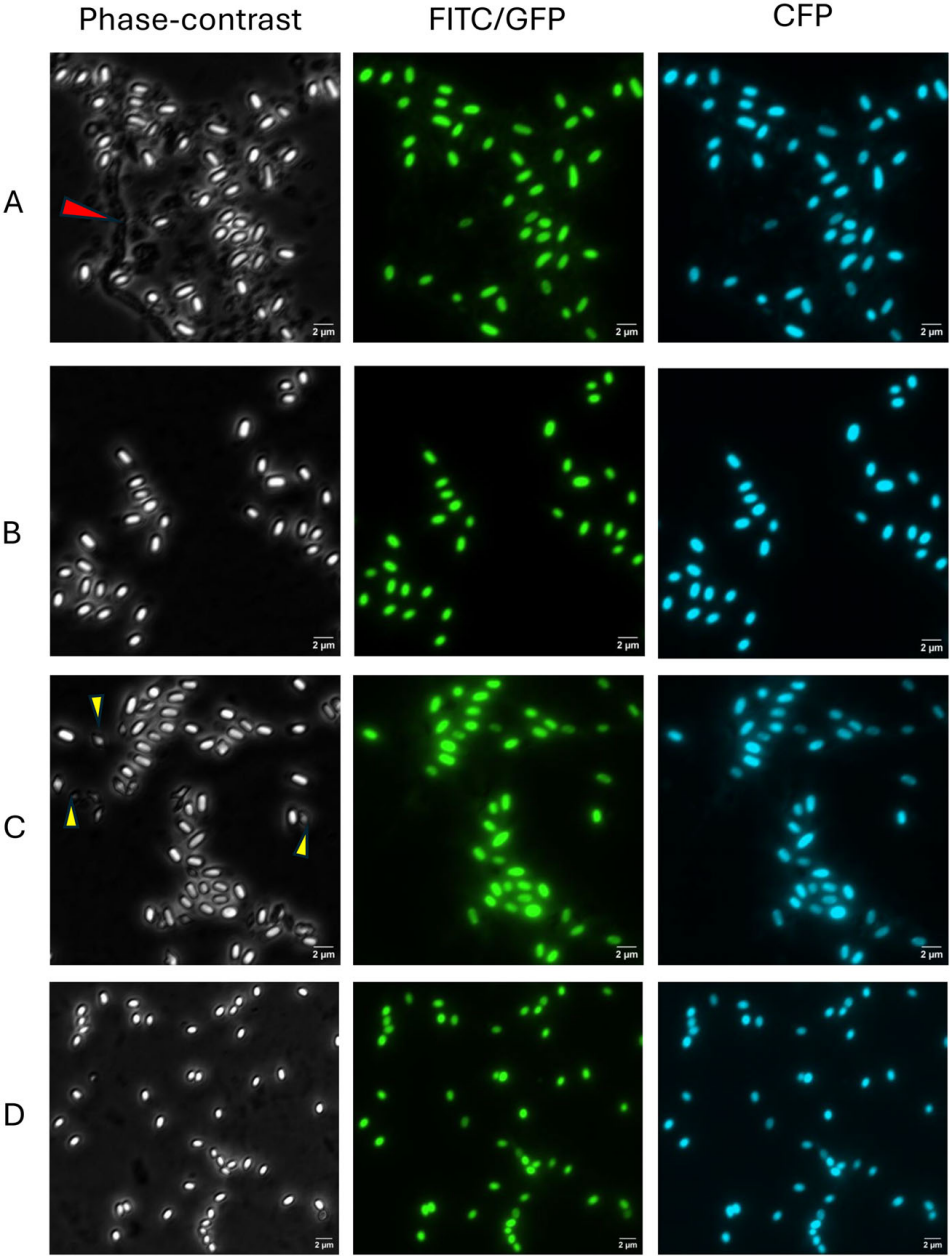
ThT‐stained fluorescence micrograph of wild‐type *Bacillus* spp. spores. (**A**) *B. paranthracis* NVH 0075/95. The red arrow indicates contaminating cell debris. (**B**) *B. thuringiensis* 407. (**C**) *B. thuringiensis* (subsp. *kurstaki*). Yellow arrows indicate co‐purifying toxin crystals. (**D**) *B. subtilis* 507. Images were acquired using 1‐s exposure times for phase‐contrast and FITC channels and 30‐ms exposure for the CFP channel. Image processing was performed using Fiji.

**Figure 7:**
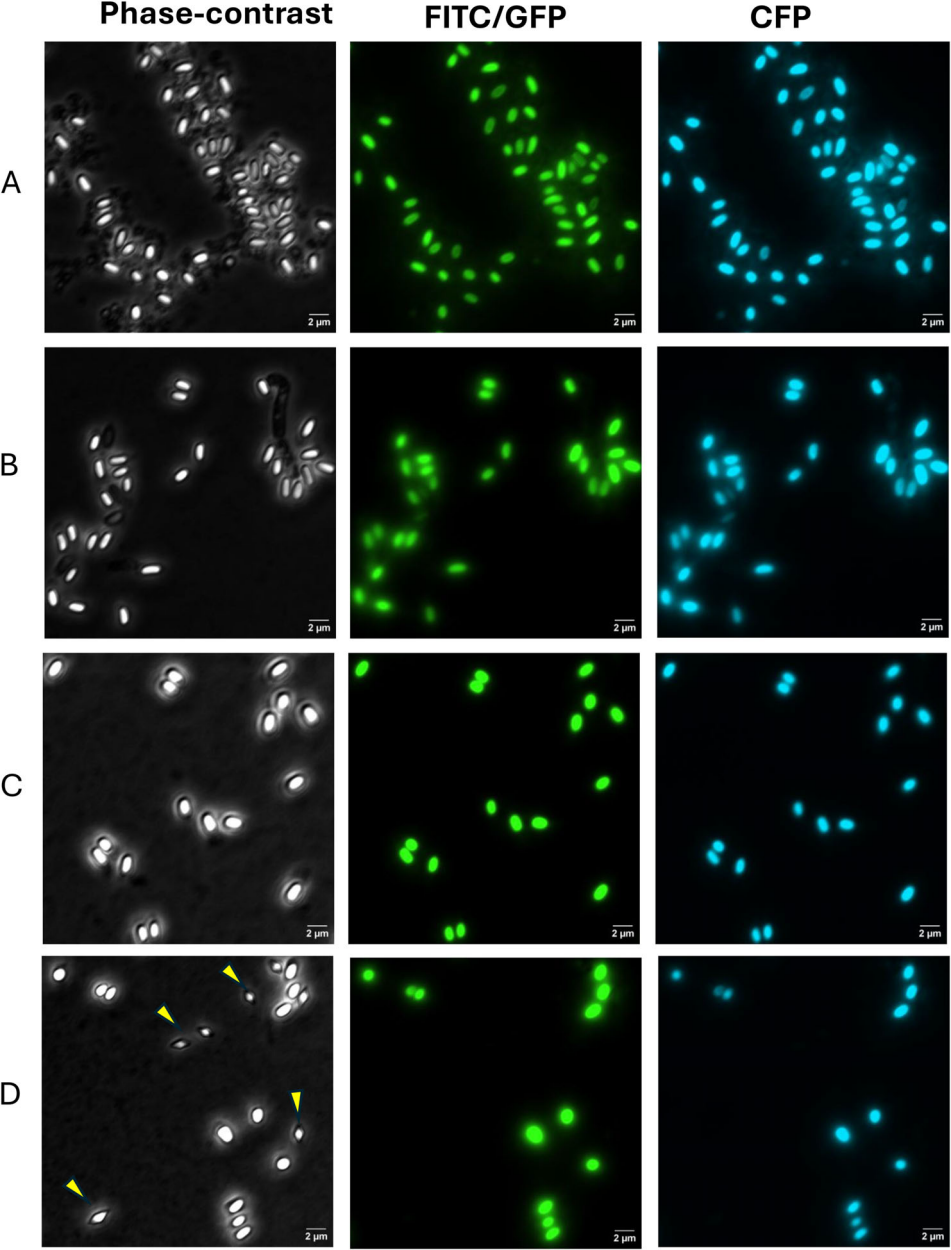
ThT‐stained ENA‐depleted and S‐ENA‐overexpressing spores. Panels (**A**), (**C**), and (**D**) show ENA‐depleted spores, whereas panel (**B**) shows S‐ENA‐overexpressing spores. (**A**) *B. paranthracis* Δ*ena1ABC*/Δ*ena3A*. (**B**) *B. paranthracis* Δ*ena1B*::p*enaAB* (S‐ENA–overexpressing strain). (**C**) *B. thuringiensis* 407 Δ*f*‐*ena*. (**D**) *B. thuringiensis* (subsp. *kurstaki*) Δ*f*‐*ena*. Yellow arrows indicate toxin crystals. Images were acquired using 1‐s exposure times for phase‐contrast and FITC channels and 30‐ms exposure for the CFP channel. Image processing was performed using Fiji.

**Figure 8:**
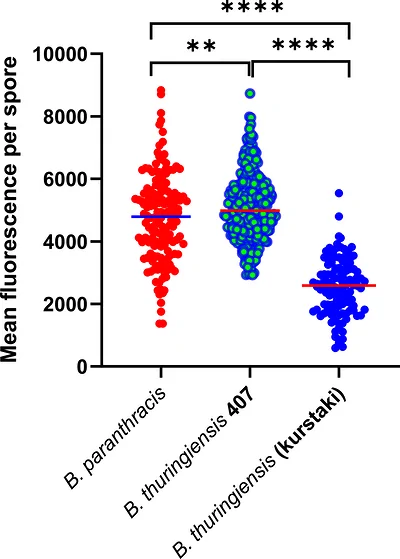
Fluorescence of ThT stained *Bacillus* spp. spores. The fluorescence intensities of individual *B. paranthracis* (*n* = 158), *B. thuringiensis* 407 (*n* = 307), and *B. thuringiensis* subsp. *kurstaki* (*n* = 114) spores were quantified from microscopy images using Fiji. Images were split into individual channels, and the CFP fluorescence channel was selected for analysis. A standardized oval region of interest (ROI; 120 × 120 pixels) was created using the “Specify” function and added to the ROI Manager. The same‐sized ROI was positioned over each spore, and the mean fluorescence intensity was obtained using the “Measure” function. Multiple blank regions containing no spores were measured to estimate background fluorescence. The measurements were exported to Microsoft Excel, and the background fluorescence was subtracted from each spore measurement. The background‐corrected mean fluorescence intensity of each spore is shown; horizontal bars indicate the median for each strain. Differences among strains were assessed using ordinary one‐way ANOVA.

Shorter staining times, e.g., a 10 min incubation of *B. paranthracis* spores with 150 µM ThT following the protocol of Xia et al. (2011), resulted in staining limited to the outer spore layers in the *B. cereus* group species we tested (see Supporting Information, Fig. S3). In contrast, incubation for 24 hr led to uniform staining of the spore (Fig. 6 and Fig. 7). As an alternative to 10 min incubation at room temperature, we evaluated heating the ThT‐spore mixture at 80°C for 10 min. Although this approach produced effective and uniform staining, it led to the appearance of a substantial number of phase‐dark spores after a few days of storage at 4°C. Notably, ThT‐stained spores retained fluorescence for at least 2.5 years when stored at 4°C (see Supporting Information, Fig. S4). In addition, ThT‐stained spores remained fluorescent after overnight fixation with 4% formaldehyde followed by two washes with water (see Supporting Information, Fig. S5). Finally, germination experiments with ThT‐stained *B. thuringiensis* spores in 96‐well plates showed that fluorescence intensity decreased as the spores transitioned from a phase‐bright to a phase‐dark state in the presence of l‐cysteine, but not l‐alanine or water (see Supporting Information, Fig. S7). These findings suggest that ThT fluorescence provides a convenient plate‐based readout for monitoring spore germination and could therefore be used in high‐throughput screening assays.

### Time Considerations

See Table 3, which outlines timing for culturing, purification, and ThT staining of *Bacillus* spp. spores

**Table 3:** Time Considerations for Culturing, Purification, and ThT staining of *Bacillus* spp. Spores

Protocol	Steps	Estimated time needed	Details
Basic Protocol 1	Spore cultivation	3‐5 weeks	Inoculation on agar plates and completion of sporulation
	Spore purification	6‐8 hr	Washing, spore enrichment, and Nycodenz gradient purification (up to 4 samples)
Basic Protocol 2	Spore weighing	30‐60 min	Centrifugation of purified spore aliquots and pellet weight measurement
	ThT solution preparation	30 min	Preparation of ThT stock and working solutions
	ThT staining	24 hr	Mixing with and incubation in ThT solution
	Fluorescence microscopy	2‐4 hr	Preparation of 1.5% agarose pad on a slide, sample application, and fluorescence imaging

### Author Contributions

**Ephrem Debebe Zegeye**: Conceptualization; data curation; formal analysis; investigation; methodology; software; validation; visualization; writing—original draft; writing—review and editing. **Marina Aspholm**: Funding acquisition; project administration; resources; supervision; validation; writing—review and editing.

### Conflict of Interest

The authors declare no conflict of interest.

## Supporting information




*Figure S1: Contaminating cellular debris adhering to the tube wall during spore washing. Figure S2: Wild‐type and F‐ENA depleted strains of* *B. thuringiensis* *407 exhibit differences during Nycodenz density gradient‐based spore purification. Figure S3: ThT‐stained* *B. paranthracis* *spores (150 µM, 10 min at room temperature) showing heterogeneity in fluorescence across the spore population, with staining restricted to the outer layers in most spores. Figure S4: ThT‐stained spores retain fluorescence for several years when stored at 4°C. Figure S5: ThT‐stained* *B. paranthracis* *spores retain fluorescence after overnight fixation in 4% paraformaldehyde (PBS) and two washes. Figure S6: ThT‐unstained spores exhibit minimal autofluorescence. Figure S7: Monitoring* *Bacillus* *spore germination using ThT fluorescence in a 96‐well plate format*.

*The complete list of* *Bacillus* *strains used in the protocol and their sources*.

## Data Availability

Raw data files are available from the authors on request.
