# Supplementary material for: Purification and Thioflavin T Staining of Bacillus spp. Endospores for Fluorescence‐Based Assays
Source: Curr Protoc. 2026 Oct 5;6(10):e70480. doi: 10.1002/cpz1.70480 (PMC13637824; doi:10.1002/cpz1.70480)
Supplement: Supplementary file 1 [file CPZ1-6-0-s001.pdf]

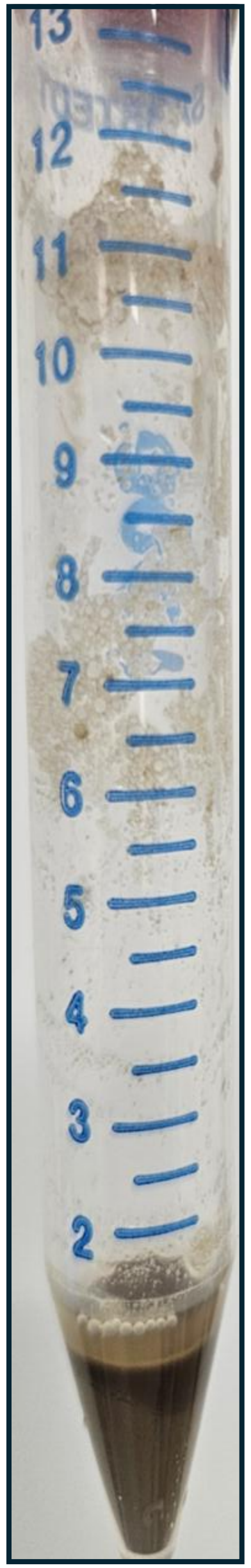

Figure S1

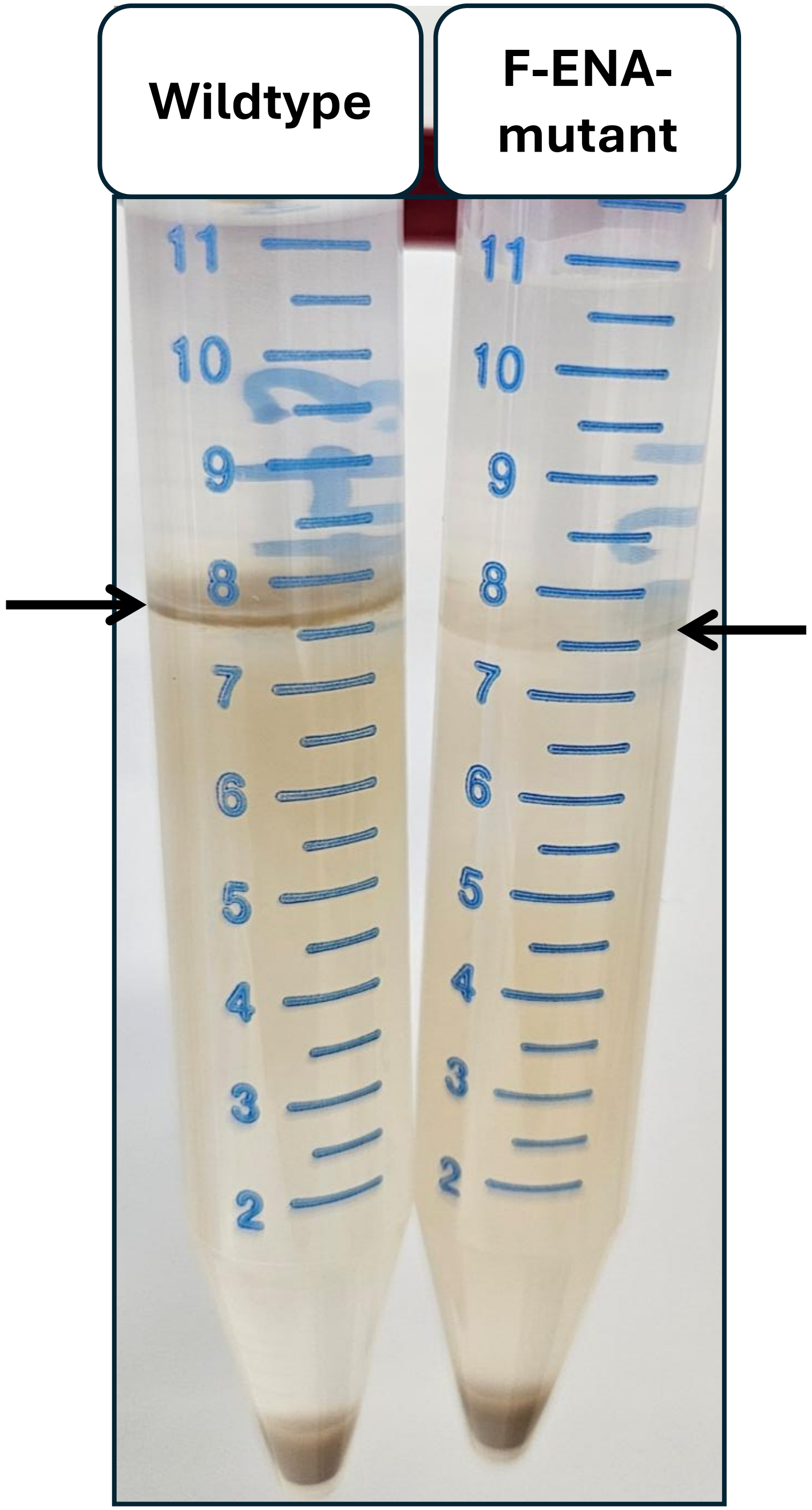

Figure S2

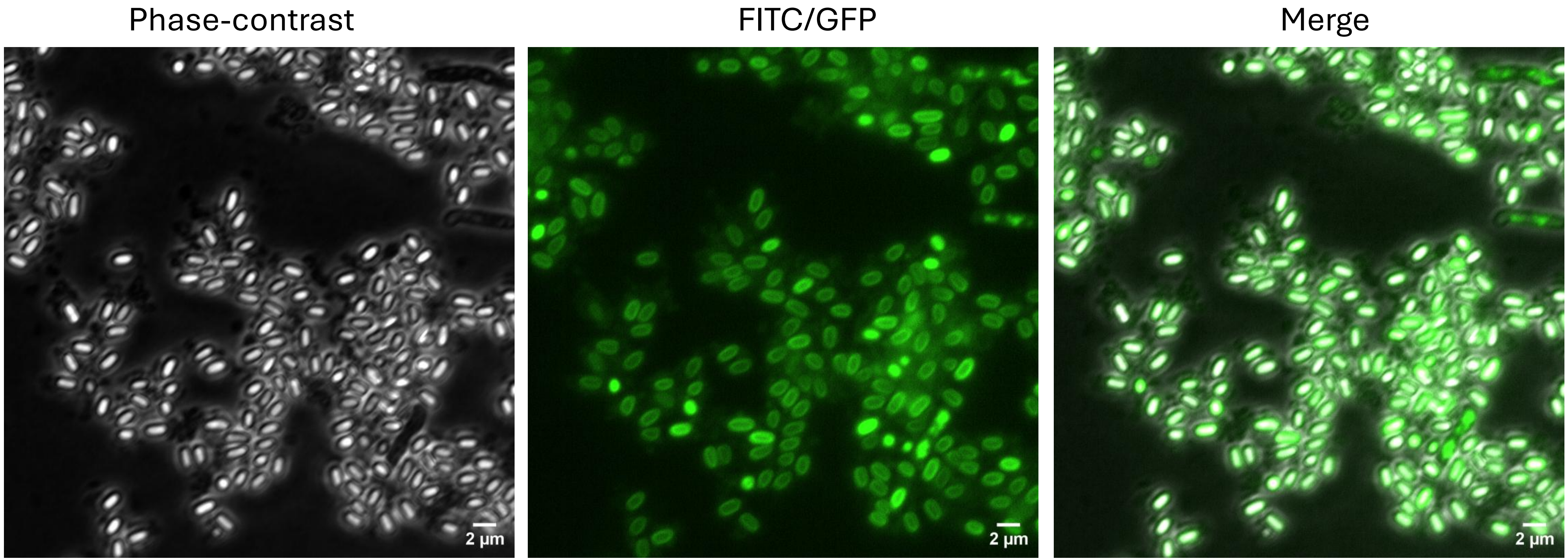

Figure S3

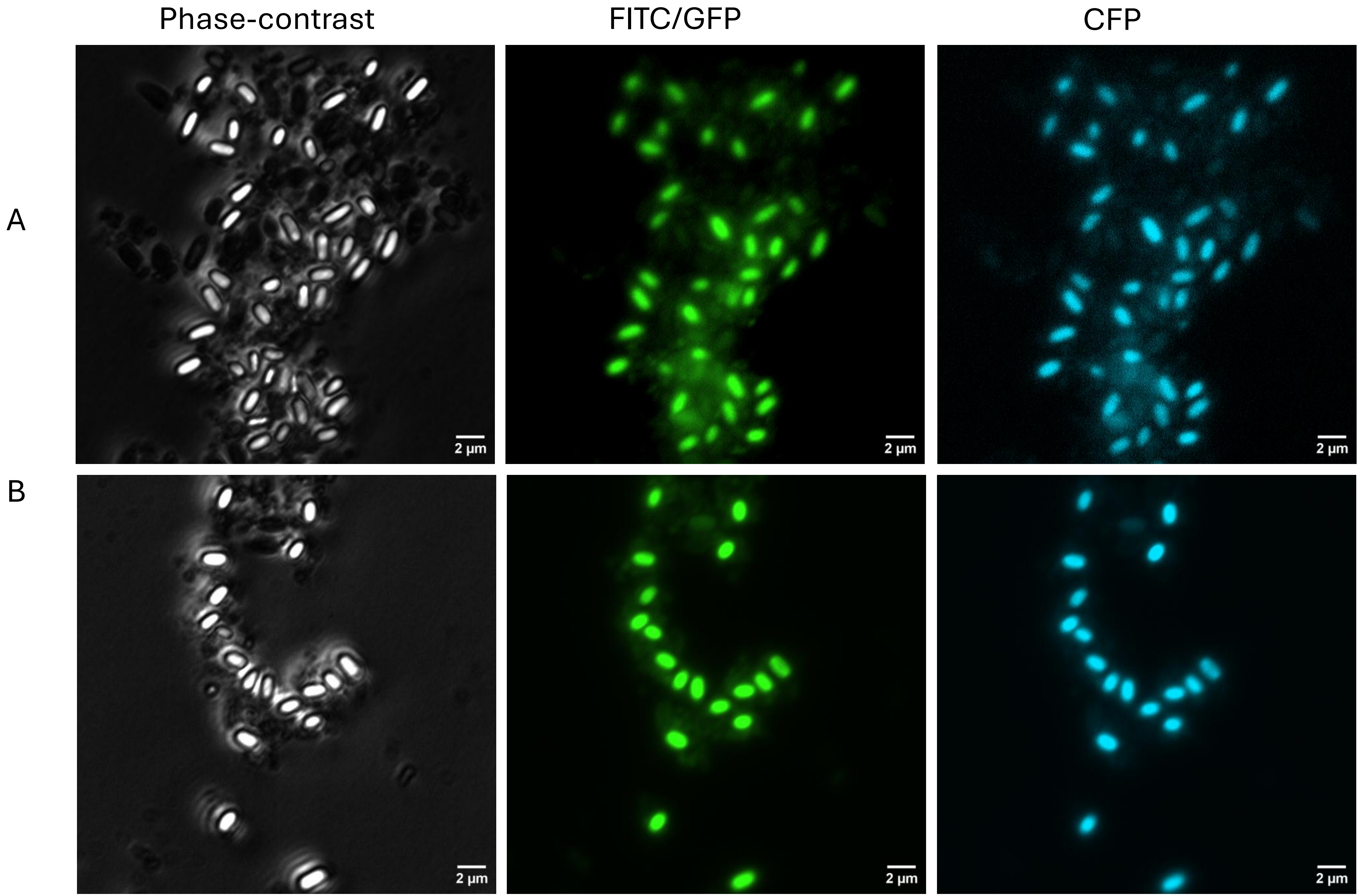

Figure S4

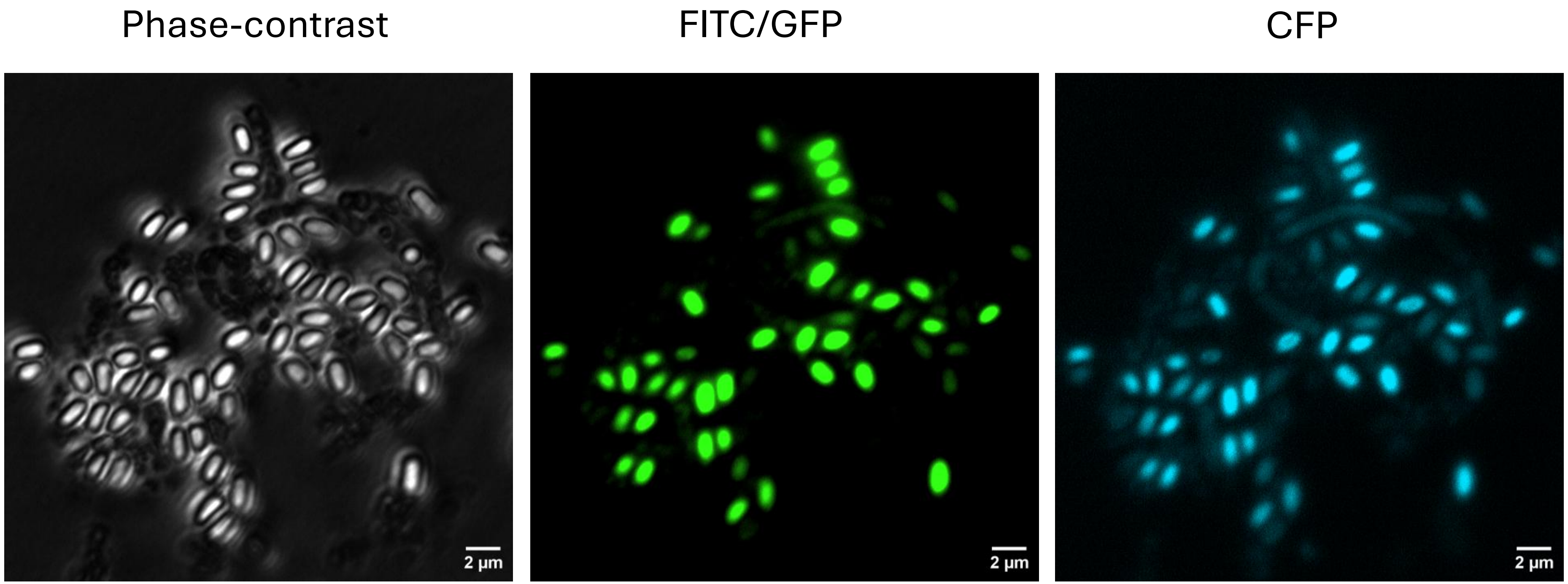

Figure S5

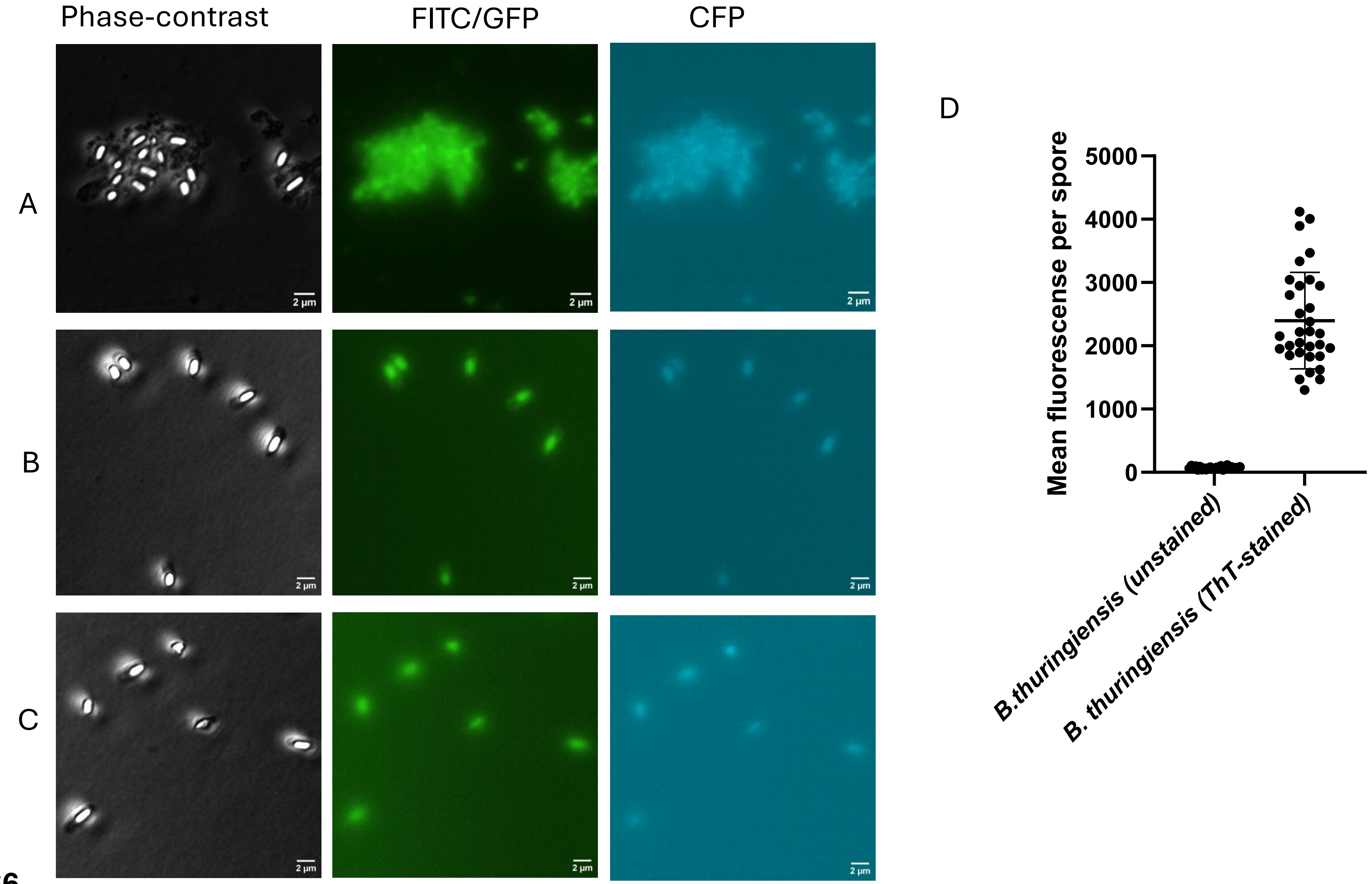
Phase-contrast
FITC/GFP
CFP
A
B
C
2 µm
D
Mean fluorescense per spore
5000
4000
3000
2000
1000
0
B.thuringiensis (unstained)
B. thuringiensis (ThT-stained)

Figure S6

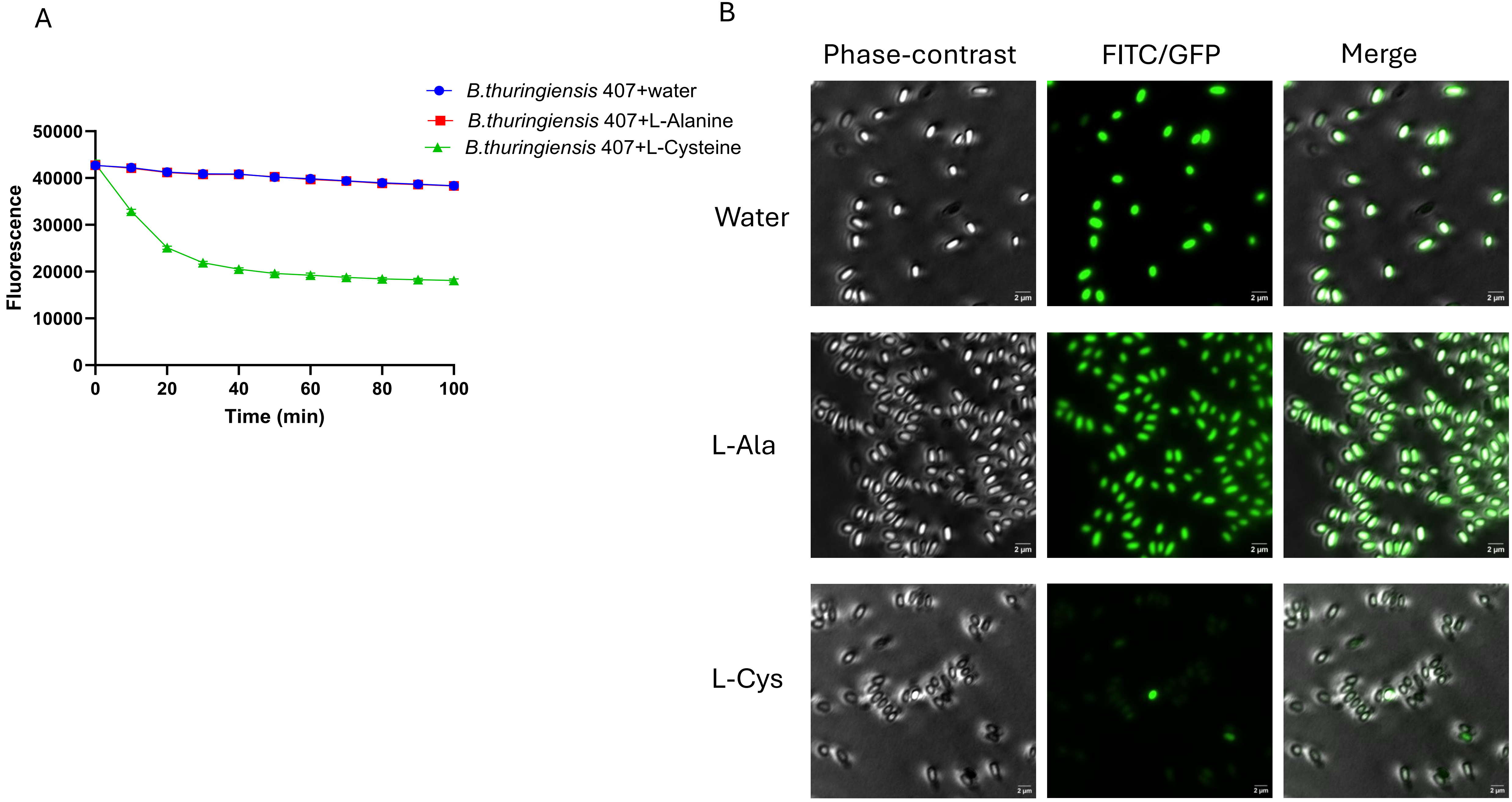

A
B.thuringiensis 407+water
B.thuringiensis 407+L-Alanine
B.thuringiensis 407+L-Cysteine
50000
40000
30000
20000
10000
0
Fluorescence
0
20
40
60
80
100
Time (min)
B
Phase-contrast
FITC/GFP
Merge
Water
L-Ala
L-Cys
2 μm

Figure S7
