# Supplementary material for: Purification and Thioflavin T Staining of Bacillus spp. Endospores for Fluorescence‐Based Assays
Source: Curr Protoc. 2026 Oct 5;6(10):e70480. doi: 10.1002/cpz1.70480 (PMC13637824; doi:10.1002/cpz1.70480)
Supplement: Supplementary file 2 [file CPZ1-6-0-s002.docx]

**Supplementary Table S1.**

| ***Strains*** | **Sources/References** |
| --- | --- |
| *Bacillus paranthracis* NVH 0075/95 | (Lund and Granum, 1996; Pradhan et al., 2021) |
| *B. paranthracis* ∆*ena1*ABC/∆*ena*3A | (Pradhan et al., 2021) |
| *B. paranthracis* ∆*ena1B*::p*enaAB* | (Pradhan et al., 2021) |
| *B. thuringiensis* 407 | (Sleutel et al., 2025) |
| *B. thuringiensis* 407 ∆*f-ena* | (Sleutel et al., 2025) |
| *B. thuringiensis* (subsp. *kurstaki*) | This study |
| *B. thuringiensis* (subsp. *kurstaki*) ∆*f-ena* | This study |
| *B. subtilis* 507 | This study |
